# Patellofemoral arthroplasty with onlay prosthesis leads to higher rates of osteoarthritis progression than inlay design implants: a systematic review

**DOI:** 10.1007/s00167-023-07404-0

**Published:** 2023-04-02

**Authors:** Manuel-Paul Sava, Georgios Neopoulos, Alexandra Leica, Michael T. Hirschmann

**Affiliations:** 1 Department of Orthopaedic Surgery and Traumatology, Kantonsspital Baselland (BruderholzLiestalLaufen), CH-4101 Bruderholz, Switzerland; 2 Department of Clinical Research, Research Group Michael T. Hirschmann, Regenerative Medicine and Biomechanics, University of Basel, CH-4001 Basel, Switzerland

**Keywords:** Patellofemoral arthroplasty, Patellofemoral replacement, PFA, PFR, Inlay, Onlay, Clinical outcomes, Functional outcomes, PROMs, Complications rate, Progression of OA, Progression of osteoarthritis, Pain, Implant survivorship, Systematic review

## Abstract

**Purpose:**

The aim of this study was to report the clinical and functional outcomes, complication rates, implant survivorship and the progression of tibiofemoral osteoarthritis (OA), after new inlay or onlay patellofemoral arthroplasty (PFA), for isolated patellofemoral OA. Comparison of different implant types and models, where it was possible, also represented one of the objectives.

**Methods:**

A systematic literature search following PRISMA guidelines was conducted on PubMed, Scopus, Embase and Cochrane databases, to identify possible relevant studies, published from the inception of these databases until 11.11.2022. Randomized control trials (RCTs), case series, case control studies and cohort studies, written in English or German, and published in peer-reviewed journals after 2010, were included. Not original studies, case reports, simulation studies, systematic reviews, or studies that included patients who underwent TKA or unicompartmental arthroplasty (UKA) of the medial or lateral compartment of the knee, were excluded. Additionally, only articles that assessed functional and/or clinical outcomes, patient-reported outcomes (PROMs), radiographic progression of OA, complication rates, implant survival rates, pain, as well as conversion to TKA rates in patients treated with PFA, using inlay or onlay trochlea designs, were included. For quality assessment, the Methodological Index for Non-Randomized Studies (MINORS) for non-comparative and comparative clinical intervention studies was used.

**Results:**

The literature search identified 404 articles. 29 of them met all the inclusion criteria following the selection process. Median MINORS for non-comparative studies value was 12.5 (range 11–14), and for comparative studies 20.1 (range 17–24). In terms of clinical and functional outcomes, no difference between onlay and inlay PFA has been described. Both designs yielded satisfactory results at short, medium and long-term follow-ups. Both designs improved pain postoperatively and no difference between them in terms of postoperative VAS has been noted, although the onlay groups presented a higher preoperative VAS. When comparing the inlay to onlay trochlea designs, the inlay group displayed a lower progression of OA rate.

**Conclusion:**

There is no difference in functional or clinical outcomes after PFA between the new inlay and the onlay designs, with both presenting an improvement in most of the scores that were used. A higher rate of OA progression was observed in the onlay design group.

**Level of evidence:**

III.

**Supplementary Information:**

The online version contains supplementary material available at 10.1007/s00167-023-07404-0.

## Introduction

Patellofemoral arthroplasty (PFA) for treatment of isolated patellofemoral osteoarthritis (OA) remains until today a controversial subject due to inconsistent results found throughout the existing literature [20, 26]. Patient selection, surgical technique, as well as implant choice have a direct effect on clinical outcomes. Historically, the first patellofemoral joint replacement was a vitallium cell patella cap designed by McKeever in 1955 [31]. Nowadays, PFA implant designs can be divided into two larger groups: inlay and onlay PFA.

First generation inlay designs, such as the Richard and Lubinus prosthesis, introduced back in 1979 [8], replaced only the worn cartilage, leaving the subchondral bone untouched. Short-term outcomes, were however not promising, with a low rate of patient satisfaction, but a high conversion rate to total knee arthroplasty (TKA) [9, 45, 47]. The second-generation, or onlay design, was introduced in the 1990s. Contrary to the first-generation inlay designs, the onlay trochlea prosthesis completely replaced the anterior compartment of the knee, providing a possibility of correcting trochlea rotation or for dysplasia [46].

Due to potential complications of onlay designs, such as patellar catching or anterior notching, overstuffing, alongside an increased bone loss when compared to inlay designs, new generation inlay trochlea implants have been introduced [18, 21, 28, 34]. These implants aim to reproduce the complex kinematics of the patellofemoral joint with less mechanical and patellofemoral complications, increased implant stability and no alteration to the soft tissue tension or extensor mechanism [11, 13, 15, 16, 41].

Up-to-date studies, which report or compare clinical or functional outcomes, complication rates, revision or conversion rates, as well as progression of OA between different trochlea designs, are limited. Hence, the aim of this study is to report the clinical and functional outcomes, complication rates, implant survivorship and the progression of the tibiofemoral OA, after inlay or onlay PFA. Comparison of different implant types and models, where it is possible, also represents one of the objectives. The extended information provided from this systematic review will help physicians improve the patients’ management, functional, clinical outcomes and, therefore, patient satisfaction.

## Materials and methods

A systematic literature search following PRISMA guidelines [37] was conducted on PubMed, Scopus, Embase and Cochrane databases to identify possible relevant studies, published from the inception of these databases until 11.11.2022. The study protocol has been registered and approved by Prospero (CRD42022330285). The search strategy can be found in Additional Material 9. Randomized control trials (RCTs), case series, case control studies and cohort studies, written in English or German, and published in peer-reviewed journals after 2010, were included in the title and abstract screening of this review. Not original studies, case reports, simulation studies, systematic reviews, or studies that included patients who underwent TKA or unicompartmental arthroplasty (UKA) of the medial or lateral compartment of the knee, were excluded. In a second step, full text analysis was performed by two authors. Articles that assessed functional and/or clinical outcomes, patient-reported outcomes (PROMs) (i.e. Knee Society Score [KSS], Oxford Knee Score [OKS], Western Ontario and McMaster Universities Arthritis Index [WOMAC], Knee Injury and Osteoarthritis Outcome Score [KOOS], American Knee Society Score [AKSS], Visual Analog scale [VAS], Hungerford and Kenna Score [HKS], International Knee Documentation Committee Score [IKDC], International Knee Society Score [IKS], Anterior Knee Pain Score [AKP], etc.), radiographic progression of OA, complication rates, implant survival rates, pain, as well as conversion to TKA rates in patients treated with PFA, using inlay or onlay trochlea designs, were included. Additionally, only articles presenting their results in numerical data form were considered. Finally, surgical technique studies, abstract only studies, studies reporting outcomes after PFA with additional UKA, robotic PFA, or reporting outcomes of the patellar components of TKA, or comparing PFA with TKA, as well as studies which did not report preoperative data, have been also excluded. In case of discrepancy regarding eligibility criteria a third author was consulted.

### Quality assessment

In order to assess the quality of the included studies, the Methodological Index for Non-Randomized Studies (MINORS) for non-comparative and comparative clinical intervention studies was used [44]. The global ideal score for non-comparative studies was 16 and for comparative studies 24. The level of evidence of each included study war also reported. With the sole purpose of improving the systematic review’s quality, articles which did not meet a score of at least 11 for non-comparative studies or at least 16 for comparative studies according to MINORS have been excluded.

### Data extraction

Title, author names, study design, year and journal of publication, abstract, level of evidence, follow-up time, design of the trochlea implant, clinical outcomes, functional outcomes, revision rates, complication rates, conversion to TKA rates, progression of OA, as well as reported pain levels and PROMs were extracted into a Microsoft Excel spreadsheet (MS Microsoft, USA).

### Statistical analysis

Continuous variables were described with means and standards deviations or medians and ranges. Categorical variables were reported with absolute and relative frequencies. A p < 0.05 was considered statistically significant.

## Results

**Fig. 1:**
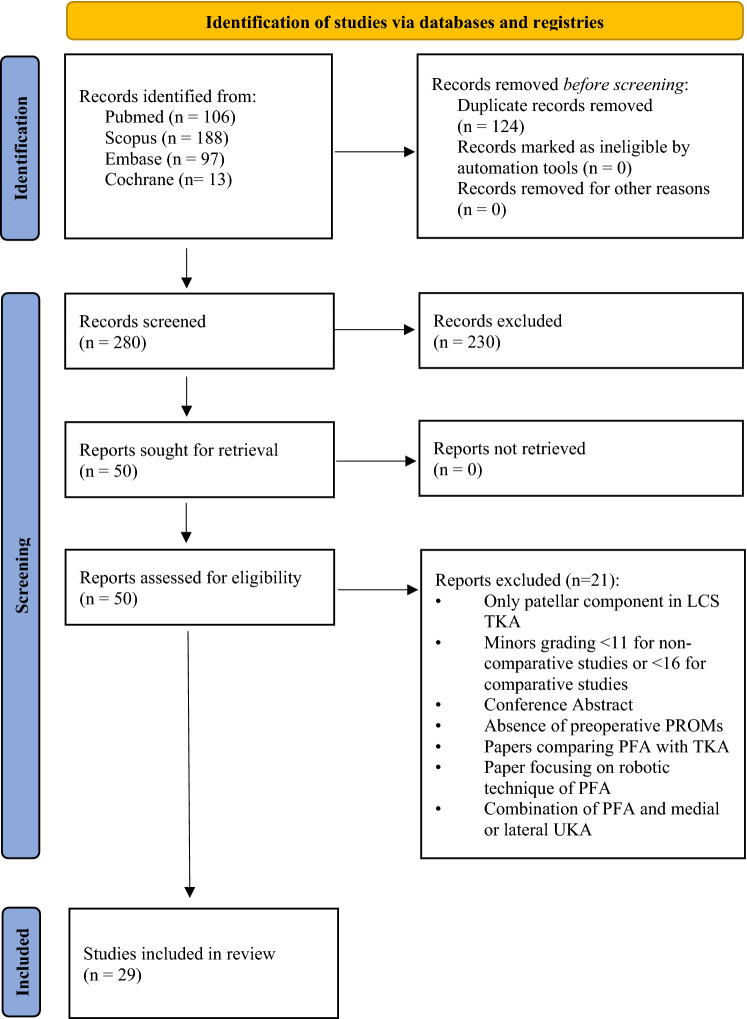
Flowchart of the study selection process according to the PRISMA 2020 statement: an updated guideline for reporting systematic reviews [37]. *LCS* low contact stress, *TKA* total knee arthroplasty, *PROMs* patient-reported outcome measures, *PFA* patellofemoral arthroplasty, *UKA* unicondylar knee arthroplasty

The literature search identified 404 publications in the initial screening process. Twenty-nine of them met all the inclusion criteria following the selection process. A detailed overview of the process is shown in Fig. 1. Median MINORS for non-comparative studies value was 12.5 (range 11–14), and for comparative studies 20.1 (range 17–24). Results from a total number of 1,761 patients were evaluated (median age at surgery 53 years, range 22–92 years). The reported median body mass index (BMI) was 26.4 (range 20–50.8). Several scores (OKS, KSS, KOOS, WOMAC, VAS, IKDC, AKP, HKS, IKS, Hospital for Special Surgery Patellofemoral Score [HSS-PF], University of California Los Angeles Score [UCLA], Short Form-36 Items [SF-36], Short Form-12 Items [SF-12], Melbourne Knee Score, Lysholm, Tegner, Kujala, Bartlett), alongside postoperative range of motion (ROM), implant survivorship, rate of complications, conversion to TKA and progression of OA, were used to evaluate clinical and functional outcomes. The detailed characteristics of the included studies are presented in Table 1.

**Table 1:** Overview of reported patients

Author (year)	Number of knees (patients)	Study type	Mean/median age, years (SD, range)	Gender male (%)	Mean/median BMI (SD, range)	Mean/median follow-up time, months (SD, range)	Level of evidence	MINORS Score
Beckmann [5]	20 knees (20 patients)	Retrospective cohort	46.4 (40–52)	nm	nm	12 (8–44)	III	17/24
Bernard [7]	153 knees (119 patients)	Retrospective cohort	55.8 (nm)	14%	29.5 (nm)	60 (± 31.2)	III	23/24
Feucht [16]	30 knees (30 patients)	Retrospective cohort	48.5 (± 8)	73%	27 (± 3)	25.5 (± 10.5)	III	24/24
Feucht [17]	41 knees (41 patients)	Retrospective cohort	48 (± 13)	39%	26 (± 3.5)	24 (nm)	III	18/24
Imhoff [22]	35 knees (34 patients)	Prospective case series	49 (± 14, 22–79)	69%	27 (± 3)	65 (± 7, 60–90)	III	14/16
Imhoff [23]	30 knees (28 patients)	Prospective cohort	42 (± 13)	52%	28 (± 3)	24 (nm)	II	18/24
Laursen [25]	18 knees (18 patients)	Retrospective case series	50 (± 12)	33%	28 (± 3.9)	24 (nm)	IV	11/16
Patel [38]	16 knees (16 patients)	Prospective case series	63 (46–83)	50%	27.2 (22.5–30)	24.1 (6–34)	III	13/16
Pogorzelski [40]	62 knees (62 patients)	Prospective case series	46 (± 11)	42%	27 (± 6)	73 (± 25)	III	14/16
Zicaro [50]	19 knees (15 patients)	Prospective case series	54 (44–65)	6%	nm	35.2 (25–54)	III	11/16
Sarda [43]	44 knees (40 patients)	Retrospective case series	61.7 (43–84)	23%	nm	54 (36–96)	IV	14/16
Mofidi [33]	34 knees (28 patients)	Retrospective case series	nm	36%	nm	12 (nm)	IV	11/16
Yadav [49]	51 knees (49 patients)	Prospective case series	54.4 (23–79)	25%	nm	50.4 (nm)	III	13/16
Beitzel [6]	22 knees (22 patients)	Prospective cohort	46.4 (± 9.3, 28–67)	64%	26.1 (± 2.6, 21.6 –30.8)	24 (nm)	II	22/24
Davies [14]	52 knees (44 patients)	Prospective case series	60.7 (38–84)	32%	nm	42 (24–60)	III	13/16
Al-Hadithy [4]	53 knees (41 patients)	Retrospective case series	62.2 (39–86)	24%	nm	37 (12–70)	IV	13/16
Akhbari [3]	61 knees (57 patients)	Prospective case series	66.1 (± 10.1)	11%	nm	61.1 (14–148)	III	14/16
Goh [19]	51 knees (51 patients)	Retrospective case series	52.7 (± 7.5, 39–72)	14%	28.7 (± 5.5, 20–43)	49.2 (26.4–73.2)	IV	13/16
Willekens [48]	35 knees (31 patients)	Retrospective case series	53 (23–105)	16%	nm	55 (23–105)	IV	12/16
Ahearn [1]	101 knees (83 patients)	Retrospective cohort	60 (26–86)	nm	nm	85 (60–105)	III	11/16
Konan [24]	51 knees (47 patients)	Prospective case series	57 (37–69)	57%	27.6 (22–34)	85.2 (60–132)	III	13/16
Osarumwense [36]	49 knees (36 patients)	Retrospective case series	59 (39–80)	36%	30 (22–41)	40 (24–58)	IV	11/16
Morris [35]	45 knees (35 patients)	Retrospective case series	55 (32–80)	14%	30.6 (18.8–50.8)	31 (12–80)	IV	13/16
Dahm [12]	61 knees (61 patients)	Retrospective case series	56 (± 10.4)	7%	30 (± 4.9)	48 (24–72)	IV	13/16
Ajnin [2]	43 knees (32 patients)	Retrospective case series	53 (42–62)	22%	34 (24–44)	64 (30–119)	IV	12/16
Metcalfe [32]	558 knees (429 patients)	Prospective case series	58.8 (25–92)	18%	nm	Minimum 24 (nm)	III	11/16
Bohu [10]	74 knees (64 patients)	Retrospective case series	59.6 (± 11.8, 31.3–82.1)	19%	nm	90 (± 85, 24–240)	IV	11/16
Rammohan [42]	103 knees (79 patients)	Retrospective case series	58 (42–78)	32%	nm	72 (24–132)	IV	13/16
Marullo [30]	120 knees (97 patients)	Retrospective cohort	66.5 (57–75)	17%	nm	73 (± 36)	IV	19/24

*BMI* body mass index (kilogram/meter^2^), *SD* standard deviation, *MINORS* methodological index for non-randomized studies, *nm* not mentioned

In terms of OKS, 13 included studies, have reported improved postoperative scores, when compared to preoperative ones [1–4, 14, 19, 24, 32, 33, 36, 38, 42, 48]. No difference was observed between inlay and onlay implants, in terms of OKS [16]. Although, a couple of studies, which do not mention *p* values or confidence intervals do exist [14, 33], the overwhelming majority of the findings qualify as statistically significant (*p* < 0.05). Patients have been followed at short, short to medium, medium and long terms. Collected data can be found in Table 2.

**Table 2:** Overview of reported OKS

Author (year)	No. of knees	Implant type	Follow-up period (months) (SD, range)	Preoperative mean/median OKS (SD, range)	Postoperative mean/median OKS (SD, range)	*P* value
Mofidi [33]	34	FPV	6 and 12 (nm)	30 (± 6)*	21 (± 12)*	nm
Davies [14]	52	FPV	12 (nm)	30.4 (16–44)*	19 (3–41)*	nm
Al-Hadithy [4]	53	FPV	12 (nm)	19.7 (4–37)**	32.1 (nm)**	< 0.05
Akhbari [3]	61	Avon	60 (nm)	20.8 (± 7.9)**	31.8 (± 8.7)**	< 0.001
Goh [19]	51	Sigma HP	24 (nm)	32.2 (± 7.8)*	22.3 (± 9.4)*	< 0.001
Willekens [48]	35	Avon	53 (23–105)	10.5 (7–14)**	32.1 (24.3–39)**	< 0.001
Ahearn [1]	101	Journey	60 (nm)	18 (nm)**	30 (21–42)**	< 0.001
Konan [24]	51	Avon	85 (60–132)	18 (5–32)**	38 (28–42)**	< 0.0005
Osarumwense [36]	49	Gender Solutions	40 (24–58)	19 (5–32)**	38 (28–42)**	< 0.0005
Patel [38]	16	HemiCap Wave	24.1 (6–34)	19 (2–30)**	35 (10–44)**	< 0.01
Ajnin [2]	43	FPV	65 (30–119)	18 (5–35)**	29 (19–45)**	0.003
Metcalfe [32]	558	Avon	180 (nm)	19 (14–25)**	35 (20–41)**	0.004
Rammohan [42]	103	Journey	60 (± 12, 24–108)	18 (15–21)**	37 (31–41)**	< 0.0001

*OKS* Oxford knee score, *SD* standard deviation, *nm* not mentioned, *FPV* Femoro-Patella Vialla, *HP* high performance, * Old OKS, ** New OKS

When discussing WOMAC, seven studies state that both inlay and onlay designs yield improved postoperative scores [1, 16, 17, 22, 23, 32, 40]. Feucht et al. also directly compared WOMAC scores, between onlay and inlay designs at a median follow-up of two years. There was no difference between the reported scores in the two groups [16]. WOMAC scores were reported at medium- and long-term follow-ups. All reported results are statistically significant (*p* < 0.05). Collected data can be found in Table 3.

**Table 3:** Overview of reported WOMAC

Author (year)	No. of knees	Implant type	Follow-up period (months) (SD, range)	Preoperative mean/median WOMAC (SD, range)	Postoperative mean/median WOMAC (SD, range)	*P* value
Imhoff [23]	30	HemiCap Wave	24 (nm)	60.6 (± 17.9)	85.2 (± 10.9)	< 0.001
Ahearn [1]	101	Journey	85 (60–105)	nm	22 (15–35)*	< 0.001
Feucht [16]	30 (15 vs. 15)	Journey and HemiCap Wave	25.5 (nm)	63 (± 14) (HemiCap Wave) 51 (± 24) (Journey)	78 (± 18) (HemiCap Wave) 78 (± 19) (Journey)	< 0.05
Metcalfe [32]	558	Avon	180 (nm)	62 (48–70)*	35 (23–45)*	0.013
Imhoff [22]	24	HemiCap Wave	60 (nm)	63 (± 18, 58–71)	74 (± 20, 68–84)	0.011
Feucht [17]	41	HemiCap Wave	24 (nm)	67.8 (± 13.6)	79.0 (± 15.3)	< 0.05
Pogorzelski [40]	62	HemiCap Wave	60 (± 25)	67 (± 16)	77 (± 19)	0.003

*WOMAC* Western Ontario and McMaster Universities osteoarthritis index, *SD* standard deviation, *nm* not mentioned, *FPV* femoro-patella vialla, *HP* high performance, * Reverse WOMAC

In the case of ROM, 12 studies were identified for data extraction. The majority of the studies reported an increase in the postoperative ROM when compared to preoperative values [1, 12, 19, 24, 27, 35, 38, 43, 50]. Contrary to the majority, Al-Hadithy et al. reported no change in ROM, when comparing preoperative values to 12-months follow-up ones [4]. Furthermore, Ajnin et al. actually reported a decrease in ROM values at a median follow-up of 65 months (range: 30–119), when compared to preoperative values [2]. No studies were found which directly compared ROM values between onlay and inlay designs. ROM was reported preoperatively and postoperatively at short-, short-to-medium-, medium and long-term follow-ups. Collected data can be found in Table 4.

**Table 4:** Overview of reported ROM

Author (year)	No. of knees	Implant type	Follow-up period (months) (SD, range)	Preoperative mean/median ROM (SD, range)	Postoperative mean/median ROM (SD, range)	*P* value
Sarda [43]	44	Avon	54 (36–96)	116º (100º-140º)	125º (100º-140º)	< 0.05
Mofidi [33]	34	FPV	6 and 12 (nm)	nm	116º (60º-130º)	nm
Al-Hadithy [4]	53	FPV	12 (nm)	120º (nm)	120º (nm)	< 0.05
Ahearn [1]	101	Journey	85 (60–105)	115º (105º-120º)	120º (115º-120º)	n.s
Konan [24]	51	Avon	85 (60–132)	116º (98º-130º)	121º (98º-129º)	nm
Liow [27]	51	Sigma HP	24 (nm)	126.6º (± 14.1º)	129.2 (± 12.1º)	n.s
Morris [35]	45 (26, 15,4)	Vanguard, Gender Solutions and Kinematch	27 (5–80)	118.6º (90º-144º)	121.8º (105º-144º)	nm
Dahm [12]	59	Avon	48 (24–72)	123º (± 9.0º)	125º (± 6.1º)	n.s
Patel [38]	16	HemiCap Wave	24.1 (6–34)	115º (nm)	120º (nm)	n.s
Ajnin [2]	43	FPV	65 (30–119)	115º (95º-130º)	110º (90º-130º)	n.s
Marullo [30]	120	Gender Solutions	84 (± 30, 24–142)	110º (110º-120º)	120º (nm, 120º-130º)	< 0.001
Goh [19]	51	Sigma HP	24 (nm)	120.6º (± 14.1º)	125.9º (± 12.1º)	n.s

Regarding KSS, almost all of the nine analysed studies reported an increase in both postoperative clinical/objective scores and functional scores, when compared to preoperative ones [5, 7, 12, 19, 30, 35, 43, 50]. Both currently circulating variants of KSS were used (KSS 1989 and KSS 2011). With the notable exceptions of Morris et al. [35], who did not mention the statistical significance and Bernard et al. [7], who did present his findings as statistically non-significant, the remaining majority of analysed studies reported their findings as statistically significant (*p* < 0.05). The KSS scores were reported preoperatively and postoperatively at short to medium, medium and long-term follow-ups. Collected data can be found in Table 5.

**Table 5:** Overview of reported KSS

Author (year)	No. of knees	Implant type	Follow-up period (months) (SD, range)	Preoperative mean/median KSS (SD, range)		Postoperative mean/median KSS (SD, range)		*P* value
				Clinical*/ Objective**	Functional	Clinical*/ Objective**	Functional	
Sarda [43]	44	Avon	54 (36–96)	nm	57 (23–95)*	nm	85 (28–100)*	< 0.05
Goh [19]	51	Sigma HP	24 (nm)	58.5 (± 19.9)**	65.9 (± 14.3)**	89.8 (± 12.0)**	82.8 (± 12.0)**	< 0.001
Osarumwense [36]	49	Zimmer Gender Solutions	40 (24–58)	nm	nm	94 (89–100)**	100 (10–100)**	< 0.0005
Morris [35]	45 (26,.15, 4)	Vanguard, Gender Solutions and Kinematch	27 (5–80)	59.4 (35–90)*	56 (29–95)*	82.4(49–100)*	62.8 (30–100)*	nm
Dahm [12]	59	Avon	48 (24–72)	51.4 (± 7.3, 37–88)**	56.0 (± 10.9, 20–70)**	89.9 (± 13.3, 57–100)**	77.6 (± 20.6, 15–100)**	0.0001
Zicaro [50]	17	HemiCap Wave	35.2 (± 13.2, 25–54)	39.8(± 13.7)*	nm	82.5(± 6.3)*	nm	< 0.0001
Beckmann [5]	20	HemiCap Wave	12 (nm)	60 (± 5.3, 60–70)* ^a^	nm	90 (± 8.3, 70–90)* ^a^	nm	0.006
Bernard [7]	153	Avon	60 (± 30)	58 (± 13.4)** ^a^	62.2 (± 23.5)** ^a^	76 (± 14.3)** ^a^	77.3 (± 23.5)** ^a^	n.s
Marullo [30]	120	Gender Solutions	84 (± 30, 24–142)	57 (52–67)*	60 (45–56)*	94 (89–99)*	90 (80–96)	< 0.001

*KSS* knee society score, *SD* standard deviation, *nm* not mentioned, *ns* not significant, *FPV* Femoro-Patella Vialla, *HP* high performance, * Old KSS (1989), ** New KSS (2011)

^a^Values from multiple groups combined into one overall group

In addition, various other PROMs were reported. AKSS was reported by three studies, at short and long-terms intervals [1, 25, 43]. They have found improved scores postoperatively when compared with preoperative ones. Tegner score was reported by five studies [7, 12, 23, 30, 40]. Furthermore, Kujala score [42, 48, 50], Lysholm score [16, 42, 50], KOOS [1, 12, 38], SF-12 and SF-36 [1, 19, 38] UCLA [12, 30], MKS [19, 43], HKS [3], IKS [10], IKDC [23], AKP [10] and HSS-PF [50] were also presented. A small difference in Lysholm score values between inlay and onlay designs has been reported at a median follow-up period of 25.5 months (range not given), with the inlay group scoring slightly higher (66 ± 11 vs. 57 ± 22) [16]. With the notable exception of Mofidi et al. [33] all other authors present their findings as statistically significant (*p* < 0.05). The collected scores were reported preoperatively and postoperatively at short to medium, medium and long-term follow-ups. Collected data can be found in Table 6.

**Table 6:** Overview of reported PROMs

Author (year)	No. of knees	Implant type	Type of score	Follow-up period (months) (SD, range)	Preoperative mean/median value (SD, range)	Postoperative mean/median value (SD, range)	*P* value
Sarda [43]	44	Avon	MKS	54 (36–96)	10 (5–21)	25 (11–30)	< 0.05
Mofidi [33]	34	FPV	AKSS total score	6 and 12 (nm)	49 (± 12)	80 (± 20)	n.s
			AKSS functional		42 (± 12)	65.5 (± 16)	n.s
Akhbari [3]	61	Avon	HKS	60 (nm)	40 (25–55)	80 (70–95)	< 0.001
Goh [19]	51	Sigma HP	MKS	24 (nm)	12.6 (± 4.6)	24.5 (± 5.8)	< 0.001
			SF-36 PCS		26.8 (± 4.7)	45.4 (± 12)	0.0001
			SF-36 MCS		45.9 (± 13)	48.7 (± 15.6)	n.s
Imhoff [23]	30	HemiCap Wave	Tegner	24 (nm)	2 (1–3)	3 (2–5)	0.005
			IKDC		41.1 (± 12.9, nm)	58.4 (± 14.9, nm)	< 0.001
Willekens [48]	35	Avon	KOOS	53 (23–105)	32.9 (25–42)	57.6 (42.3–72.5)	< 0.001
			Kujala		35 (27.5–44)	55 (40.3–73.3)	< 0.001
Ahearn [1]	101	Journey	AKSS pain	85 (60–105)	nm	33 (20–50)	< 0.001
			AKSS functional		nm	63 (45–85)	0.002
			SF-12 PCS		nm	33.8 (31.2–36.4)	nm
			SF-12 MCS		nm	45.3 (42.9–47.7)	nm
Feucht [16]	30 (15 vs. 15)	Journey and HemiCap Wave	Lysholm	25.5 (nm)	34 (± 11) (HemiCap Wave) 32 (± 20) (Journey)	66 (± 11) (HemiCap Wave) 57 (± 22) (Journey)	< 0.05
Laursen [25]	18	HemiCap Wave	AKSS clinical	12 (nm)	49.4 (± 4.5)	85.3 (± 8.7)	< 0.01
			AKSS functional		50 (± 4.5)	87.8 (± 7.7)	< 0.01
Dahm [12]	59	Avon	Tegner	48 (24–72)	2.3 (± 0.9, 0–4)	3.8 (± 1.2, 0–5)	0.0001
			UCLA		3.4 (± 0.5, 2–5)	5.8 (± 1.8, 2–9)	0.0001
Patel [38]	16	HemiCap Wave	KOOS	24.1 (6–34)	39 (5–64)	55 (33–85)	< 0.01
			SF-36 PCS		32 (19–40)	53 (19–70)	< 0.01
			SF-36 MCS		42 (18–55)	45 (20–62)	n.s
Zicaro [50]	17	HemiCap Wave	Lysholm	35.2 (± 13.2, 25–54)	31.9 (± 14.5)	85.8 (± 9.0)	< 0.0001
			Kujala		32.1 (± 17.5)	79.3 (± 10.7)	< 0.0001
			HSS-PF		15.9 (± 15.4)	90.6 (± 6.6)	< 0.0001
Ajnin [2]	43	FPV	Kujala	65 (30–119)	35 (15–74)	58 (24–91)	0.002
Bohu [10]	30	Hermes	IKS	240 (nm)	36.3 (± 11.8)	42.3 (± 22.1)	0.03
			AKP		47.2 (± 17.8)	72.5 (± 14.6)	< 0.0001
Rammohan [42]	103	Journey	Lysholm	60 (± 12, 24–108)	27 (20–42)	81 (60–89)	0.0008
			Kujala		33 (23.5–42.5)	63.5 (44.3–78.5)	0.0009
			Modified Tegner		Level 2	Level 3	0.023
			Bartlett		13 (9–14)	25 (18–30)	0.0002
Bernard [7]	153	Avon	Tegner	60 (± 30)	2 (± 1)^a^	4 (± 1)^a^	n.s
Pogorzelski [40]	62	HemiCap Wave	Tegner	60 (± 25)	3 (± 2)	4 (± 1)	< 0.001
Marullo [30]	120	Gender Solutions	UCLA	84 (± 30, 24–142)	3 (2–4)	5 (3–7)	< 0.001
			Tegner		2 (1–2)	3 (2–3)	< 0.001

*PROMs* patient reported outcome measures, *SD* standard deviation, *nm* not mentioned, *n.s.* not significant, *MKS* Melbourne knee score, AKSS American knee society score, *HKS* Hungerford and Kenna score, *PROMs* patient reported outcomes, *SF-36* short form-36 items, *SF-12* short form-12 items, *PCS* physical component score, *MCS* mental component score, *IKDC* international knee documentation committee score, *KOOS* knee injury and osteoarthritis outcome score, *UCLA* University of California Los Angeles, *HSS-PF* hospital for special surgery patellofemoral score, *IKS* international knee society score, *AKP* anterior knee pain score

^a^Values from multiple groups combined into one overall group

Nine out of ten identified studies have reported a statistically significant reduction in perceived pain (*p* < 0.05) [5, 16, 22, 23, 25, 30, 40, 48, 50]. When comparing onlay designs with inlay ones, Feucht et al. showed that although both groups exhibited the same mean postoperative VAS value (4 ± 3), the mean preoperative VAS value was much higher in the onlay group (8 ± 2), when compared to the inlay group (6 ± 2) [16]. Scores have been reported preoperatively and postoperatively at short, short to medium, medium and long-term follow-ups. Collected data can be found in Table 7.

**Table 7:** Overview of reported VAS

Author (year)	No. of knees	Implant type	Follow-up period (months) (SD, range)	Preoperative mean/median VAS (SD, range)	Postoperative mean/median VAS (SD, range)	*P* value
Imhoff [23]	30	HemiCap Wave	24 (nm)	6.2 (± 2)	3.1 (± 2.4)	< 0.001
Willekens [48]	35	Avon	53 (23–105)	7.6 (6.7–8.5)	4.1 (2.3–5.8)	< 0.001
Konan [24]	51	Avon	85 (60–132)	nm	8 (7–9)	< 0.001
Laursen [25]	18	HemiCap Wave	12 (nm)	7.5 (± 0.8)	3.8 (± 1.3)	< 0.01
Zicaro [50]	17	HemiCap Wave	35.2 (± 13.2, 25–54)	8 (± 0.9)	2.5 (± 1.9)	0.000
Beckmann [5]	20	HemiCap Wave	12 (nm)	7 (± 0.8, 6–8)	2 (± 0.8, 1–4)	< 0.001
Imhoff [22]	24	HemiCap Wave	60 (nm)	6 (± 2, 5–7)	3 (± 3, 2–4)	< 0.001
Feucht [16]	30 (15 vs. 15)	Journey and HemiCap Wave	25.5 (nm)	6 (± 2) (HemiCap Wave) 8 (± 2) (Journey)	4 (± 3) (HemiCap Wave) 4 (± 3) (Journey)	< 0.05
Pogorzelski [40]	62	HemiCap Wave	60 (± 25)	6 (± 2)	3 (± 2)	< 0.001
Marullo [30]	120	Gender Solutions	84 (± 30, 24–142)	8 (7–9)	2 (1–3)	< 0.001

*VAS* visual analog scale, *SD* standard deviation, *nm* not mentioned

In the case of reported complications, complication rates and implant survivorship, the present findings tend to exhibit a high degree of heterogeneity. In total, 19 studies were identified [1–3, 5, 7, 10, 19, 22, 24, 25, 30, 32, 36, 38, 40, 42, 43, 49, 50]. Complication rates varied greatly among analysed studies, from as low as 0.0% [25, 36] to as high as 35.3% [49] or even 41.2% [50]. The most commonly reported complication was patellar maltracking, followed closely by anterior knee pain [1–3, 5, 7, 10, 19, 22, 24, 30, 32, 38, 42, 43, 49, 50]. Reported revision rates also exhibited an elevated degree of heterogeneity between them, with some studies stating low revision rates of 3.8% [42] or 3.9% [24], and others reporting high revision rates such as 50.0% [22] or even 55.0% [5]. No studies directly compared the type and rate of complications, or the rate of revisions between onlay and inlay type of prostheses. Results were reported postoperatively at short-to-medium-, medium- and long-term follow-ups. Collected data can be found in Table 8.

**Table 8:** Overview of reported complications and revision rate

Author (year)	No. of knees	Implant type	Follow-up period (months) (SD, range)	Complication rate (%)	Type of complication	Revision rate (%)
Sarda [43]	44	Avon	54 (36–96)	10 (20.5%)	Patellar maltracking; Anterior knee pain	4 (9.1%)
Yadav [49]	51	LCS	54.4 (23–79)	18 (35.3%)	Patellar maltracking	10 (19.6%)
Akhbari [3]	61	Avon	120 (nm)	nm	Patellar maltracking	4 (6.6%)
Goh [19]	51	Sigma HP	49 (26–73)	nm	Patellar maltracking; Anterior knee pain	4 (7.8%)
Ahearn [1]	101	Journey	85 (60–105)	7 (7.1%)	Patellar maltracking Anterior knee pain Superficial wound infection; Deep wound infection; Broken trochlear component	12 (11.9%)
Konan [24]	51	Avon	85 (60–132)	1 (2.0%)	Anterior knee pain	2 (3.9%)
Laursen [25]	18	HemiCap Wave	72 (nm)	0 (0.0%)	nm	5 (27.8%)
Osarumwense [36]	49	Zimmer Gender Solutions	40 (24–58)	0 (0.0%)	nm	2 (4.1%)
Patel [38]	16	HemiCap Wave	24.1 (6–34)	3 (18.8%)	Deep wound infection; Keloid scaring; Synovitis	1 (6.3%)
Zicaro [37]	17	HemiCap Wave	35.2 (±13.2, 25–54)	7 (41.2%)	Anterior knee pain; Patellar maltracking; ITB syndrome Joint stiffness; Non-union of the TAT	2 (11.8%)
Metcalfe [32]	558	Avon	180 (nm)	nm	Anterior knee pain; Femoral loosening; Button wear; Patellar maltracking; Avascular necrosis of the femoral condyle	105 (18.8%)
Ajnin [2]	43	FPV	65 (30–119)	11 (25.6%)	Anterior knee pain; Joint stiffness; Superficial knee infection	6 (13.9%)
Beckmann [5]	20	HemiCap Wave	29 (21–42)	nm	Anterior knee pain; Patellar malltracking	11 (55.0%)
Bohu [10]	30	Hermes	240 (nm)	nm	Patellar malltracking	10 (33.3%)
Imhoff [22]	24	HemiCap Wave	60 (nm)	6 (25.0%)	Anterior knee pain; Synovitis; Component disassembly	12 (50.0%)
Rammohan [42]	103	Journey	60 (±12, 24–108)	13 (12.6%)	Anterior knee pain; Patellar malltracking; Meniscal tear; Superficial knee infection; Haematoma; Patellar fracture	4 (3.9%)
Bernard [7]	153	Avon	60 (±30)	5 (3.3%)	Deep wound infection; Synovitis; Patellar maltracking; Patellar fracture; Deep vein thrombosis	10 (6.5%)
Pogorzelski [40]	62	HemiCap Wave	60 (±25)	nm	nm	14 (22.6%)
Marullo [30]	120	Gender Solutions	84 (±30, 24–142)	nm	Patellar maltracking; Infection; Haemarthrosis	9 (7.5%)

In terms of progression of OA and conversion to TKA, an elevated variance in findings has been noted between the 23 analysed studies [1–4, 6, 7, 10, 12, 16, 19, 22–25, 30, 32, 36, 38, 40, 42, 43, 48, 49]. Reported rates of OA progression varied from as low as 0.0% [22, 23] and 3.9% [24, 49] at 24 and 60 months follow-up, up to 32.2% [12] and even 53.3% [16] at a median follow-up period of 48 months (range: 24–72 months) and 25.5 months (range not given) follow-ups. When comparing the rate of OA progression between inlay and onlay designs, Feucht et al. found a notable difference, with the reported rate of OA progression being 0.0% in the inlay group and 53.3% in the onlay group [16]. In the case of conversion to TKA, the reported rates of conversion vary from 0.0% [38] and 0.8% [30], at a reported median of 24.1 months (range: 6–34 months) and 84 months (range: 24–142 months) follow-ups, up to 27.8% [25] and 30.0% [10], at 6 and 20 years follow-ups. No difference in reported rates of conversion to TKA has been noted [16]. Results were analysed at short-, short-to-medium-, medium- and long-term follow-ups. Collected data can be found in Table 9.

**Table 9:** Overview of reported progression of OA

Author (year)	No. of knees	Implant type	Follow-up period (months) (SD, range)	Progression of OA (%)	Conversion to TKA rate (%)
Sarda [43]	44	Avon	54 (36–96)	3 (6.8%)	1 (2.3%)
Yadav [49]	51	LCS	54.4 (23–79)	2 (3.9%)	5 (9.8%)
Beitzel [6]	22	Journey	24 (nm)	nm	1 (4.5%)
Al-Hadithy [4]	53	FPV	12 (nm)	6 (11.3%)	2 (3.8%)
Akhbari [3]	61	Avon	120 (nm)	3 (4.9%)	3 (4.9%)
Goh [29]	51	Sigma HP	49 (26–73)	2 (4.0%)	2 (4.0%)
Imhoff [23]	30	HemiCap Wave	24 (nm)	0 (0.0%)	1 (3.3%)
Willekens [48]	35	Avon	53 (23–105)	10 (28.6%)	3 (8.6%)
Ahearn [1]	101	Journey	85 (60–105)	8 (7.9%)	8 (7.9%)
Konan [23]	51	Avon	85 (60–132)	2 (3.9%)	2 (3.9%)
Feucht [16]	30 (15 vs. 15)	Journey and HemiCap Wave	25.5 (nm)	Journey 8 (53.3%) HemiCap Wave 0 (0.0%)	Journey 1 (6.7%) HemiCap Wave 1 (6.7%)
Laursen [25]	18	HemiCap Wave	72 (nm)	5 (27.8%)	5 (27.8%)
Osarumwense [36]	49	Zimmer Gender Solutions	40 (24–58)	2 (4.1%)	2 (4.1%)
Dahm [12]	59	Avon	48 (24–72)	19 (32.2%)	2 (3.4%)
Patel [38]	16	HemiCap Wave	24.1 (6–34)	1 (6.3%)	0 (0.0%)
Metcalfe [32]	558	Avon	180 (nm)	61 (10.9%)	61 (10.9%)
Ajnin [2]	43	FPV	65 (30–119)	5 (11.6%)	6 (13.9%)
Bohu [10]	30	Hermes	240 (nm)	9 (30.0%)	9 (30.0%)
Imhoff [22]	24	HemiCap Wave	60 (nm)	0 (0.0%)	6 (25.0%)
Rammohan [42]	103	Journey	60 (± 12, 24–108)	9 (8.7%)	2 (1.9%)
Bernard [7]	153	Avon	60 (± 30)	9 (5.9%)	9 (5.9%)
Pogorzelski [40]	62	HemiCap Wave	60 (± 25)	12 (19.4%)	12 (19.4%)
Marullo [30]	120	Gender Solutions	84 (± 30, 24–142)	5 (4.2%)	1 (0.8%)

*OA* osteoarthritis, *SD* standard deviation, *nm* not mentioned, *TKA* total knee arthroplasty

## Discussion

The main finding of the present review was that both onlay and inlay PFA yield satisfactory clinical and functional outcomes at short-, medium- and long-term follow-ups. No difference between designs has been described, although only one study from Feucht et al. [16] directly compared onlay and inlay designs using WOMAC and Lysholm scores, which presented a small and statistically non-significant difference in favour of the inlay design. Both designs improved pain postoperatively and no difference between them in terms of postoperative VAS has been noted, although the onlay group presented a higher preoperative VAS [16].

Regarding complication rates, implant survivorship and revision rates, the studies presented a high degree of heterogeneity between them. The most common complication described was the patella maltracking, followed closely by anterior knee pain.

One interesting finding of the study pertains to the progression of OA in the tibiofemoral compartment. When comparing the inlay and onlay trochlea designs Feucht et al. [16] found a statistically significant difference, in favour of the inlay group.

There are several systematic reviews in the literature, which report on PROMs and survivorship of the patellofemoral arthroplasty [29, 39, 46]. However, this is the first systematic review, which undertakes such a comprehensive analysis of postoperative outcomes. Additionally, none of them presents the results of both onlay designs and the new inlay designs. Pisanu et al. noted satisfactory results at short to mid-term follow-ups, and a 10 years survivorship of 90% with onlay designs, whereas inlay type of prosthesis showed disappointing results, with high rates of complications and failures [39]. This might be due the fact that included studies had reported results of first generation inlay designs only.

The systematic review of Lonner et al. based on the Australian National Joint Registry also described a 5-year cumulative revision rate of more than 20% in the case of inlay, and less than 10% when discussing onlay [29]. This is also because only first generation inlay designs were analysed. Progression of tibiofemoral OA after a successful PFA was found as the most common reason for failure [29].

This study has several limitations. First of all, the lack of more than one available studies in the literature, which directly compared the new inlay type of trochlea prosthesis, with the onlay design. Furthermore, no available RCTs pertaining to this subject have been found in the current literature. Another weakness is the retrospective type of the majority of the included studies, which could have led to an unknown selection bias. Another important aspect is that there are no studies reporting at mid- and long-term follow ups regarding the new inlay type of prosthesis, meaning that safe conclusions, with regards to the clinical and functional outcomes, and the survivorship of this type of prosthesis should be drawn with all due caution. Lastly, many authors were consultants for the companies designing the type of prostheses investigated in the respective studies, which might have led to a conflict of interest.

This systematic review provides physicians with valuable information to improve patient management, functional and clinical outcomes, and increase patient satisfaction.

## Conclusion

There is no difference in functional or clinical outcomes after PFA between the new inlay and the onlay designs, with both presenting an improvement in most of the scores that were used. A higher rate of OA progression was observed in the onlay design group.

## Supplementary Information

Below is the link to the electronic supplementary material.

Supplementary file1 (DOCX 107 KB)

## Data Availability

Data is stored on personal storage and available on request.

## Abbreviations

AKP: Anterior knee pain score
AKSS: American knee society score
BMI: Body mass index
HKS: Hungerford and Kenna score
HSS-PF: Hospital for special surgery patellofemoral score
IKDC: International knee documentation committee score
IKS: International knee society score
KOOS: Knee injury and osteoarthritis outcome score
KSS: Knee society score
MINORS: Methodological index for non-randomized studies
OA: Osteoarthritis
OKS: Oxford knee score
PFA: Patellofemoral arthroplasty
PF-CAT: Physical function-computerized adaptive test
PROMs: Patient-reported outcomes measures
RCT: Randomized control trial
ROM: Range of motion
SF-12: Short form-12 items
SF-36: Short form-36 items
TKA: Total knee arthroplasty
UCLA: University of California Los Angeles score
UKA: Unicondylar knee arthroplasty
VAS: Visual analog scale
WOMAC: Western Ontario and McMaster Universities arthritis index
