# Supplementary material for: Patellofemoral arthroplasty with onlay prosthesis leads to higher rates of osteoarthritis progression than inlay design implants: a systematic review
Source: Knee Surg Sports Traumatol Arthrosc. 2023 Apr 2;31(9):3927–40. doi: 10.1007/s00167-023-07404-0 (PMC10435614; doi:10.1007/s00167-023-07404-0)
Supplement: Supplementary file 1 — Supplementary file1 (DOCX 107 KB) [file 167_2023_7404_MOESM1_ESM.docx]

**Scopus**

(TITLE-ABS-KEY ("Clinical outcomes" OR "Functional outcomes" OR "Patient* reported outcomes" OR "Patient satisfaction" OR "Result*" OR "Risk of revision" OR "Survival" OR "Reoperation" OR "Range of motion" OR "ROM" OR "PROM" OR "PROMs" OR "Progression of osteoarthr*" OR "Progression of OA" OR "Radiological result*")) AND (TITLE-ABS-KEY ("patellofemoral arthroplasty" OR "patellofemoral prosthesis" OR "patellofemoral replacement" OR "patellofemoral joint" OR "patellofemoral arthritis" OR "patellofemoral osteoarthritis" OR "patellofemoral OA" OR "femoropatellar arthroplasty" OR "femoropatellar replacement" OR "femoropatellar prosthesis" OR "femoropatellar joint" OR "femoropatellar arthritis" OR "femoropatellar osteoarthritis" OR "femoropatellar OA" OR "pfa" OR "pfr")) AND (TITLE-ABS-KEY ("inlay" OR "onlay" OR "inlay design" OR "onlay design" OR "second generation" OR "avon" OR "zimmer" OR "journey" OR "hermes" OR "fpv" OR " lcs" OR "sigma" OR "vanguard" OR "leicester and gender" OR "hemicap wave" OR "arthrosurface")) AND ( LIMIT-TO ( SUBJAREA,"MEDI" ) )

***188 results***

**Pubmed:**

("Treatment Outcome"[MeSH Terms] OR "Patient Reported Outcome Measures"[MeSH Terms] OR "range of motion, articular"[MeSH Terms] OR "Reoperation"[MeSH Terms] OR "Patient Satisfaction"[MeSH Terms] OR "outcome*"[Text Word] OR "clinical outcome*"[Text Word] OR "radiological result*"[Text Word] OR "PROM"[Text Word] OR "PROMs"[Text Word] OR "patient reported outcome*"[Text Word] OR "Satisfaction"[Text Word] OR "result*"[Text Word] OR "ROM"[Text Word] OR "Range of motion"[Text Word] OR "Range of movement"[Text Word] OR "Risk of revision"[Text Word] OR "Survival"[Text Word]) AND ("patellofemoral joint"[MeSH Terms] OR "patellofemoral joint"[Text Word] OR "Patellofemoral Arthroplasty"[Text Word] OR "Patello-femoral joint"[Text Word] OR "patellofemoral joint"[Text Word] OR "PFA"[Text Word] OR "Patello-femoral Osteoarthritis"[Text Word] OR "Patellofemoral Osteoarthritis"[Text Word] OR "Patello-femoral OA"[Text Word] OR "Patellofemoral OA"[Text Word]) AND ("Inlays"[MeSH Terms] OR "Inlay"[Text Word] OR "Onlay"[Text Word] OR "inlay design*"[Text Word] OR "onlay design*"[Text Word] OR "second generation" [Text Word] OR "AVON"[Text Word] OR "Hermes"[Text Word] OR "Journey"[Text Word] OR "Zimmer"[Text Word] OR "LCS"[Text Word] OR "Hemicap Wave"[Text Word] OR "Sigma"[Text Word] OR "Vanguard"[Text Word] OR "Leicester and gender"[Text Word] OR "Arthrosurface"[Text Word])

***106 results***

**Cochrane:**

All text: ("Clinical outcomes" OR "Functional outcomes" OR "Patient* reported outcomes" OR "Patient satisfaction" OR "Result*" OR "Risk of revision" OR "Survival" OR "Reoperation" OR "Range of motion" OR "ROM" OR "PROM" OR "PROMs" OR "Progression of osteoarthr*" OR "Progression of OA" OR "Radiological result*") AND ("patellofemoral arthroplasty" OR "patellofemoral prosthesis" OR "patellofemoral replacement" OR "patellofemoral joint" OR "patellofemoral arthritis" OR "patellofemoral osteoarthritis" OR "patellofemoral OA" OR "femoropatellar arthroplasty" OR "femoropatellar replacement" OR "femoropatellar prosthesis" OR "femoropatellar joint" OR "femoropatellar arthritis" OR "femoropatellar osteoarthritis" OR "femoropatellar OA" OR "pfa" OR "pfr") AND ("inlay" OR "onlay" OR "inlay design" OR "onlay design" OR "second generation" OR "avon" OR "zimmer" OR "journey" OR "hermes" OR "fpv" OR " lcs" OR "sigma" OR "vanguard" OR "leicester and gender" OR "hemicap wave" OR "arthrosurface")

***13 Results (1 Review and 12 Trials)***

**Embase:**

All fields: (('outcome'/exp OR 'outcome' OR 'clinical outcome'/exp OR 'clinical outcome' OR 'radiodiagnosis'/exp OR 'radiodiagnosis' OR 'patient-reported outcome'/exp OR 'patient-reported outcome') AND ('satisfaction score'/exp OR 'satisfaction score') OR 'range of motion'/exp OR 'range of motion' OR 'joint characteristics and functions'/exp OR 'joint characteristics and functions') AND ([english]/lim OR [german]/lim) AND ('patellofemoral arthroplasty'/exp OR 'patellofemoral arthroplasty' OR 'patellofemoral prosthesis'/exp OR 'patellofemoral prosthesis')

***97 results***
